# The risk of osteomyelitis after mandibular fracture is doubled in men versus women: analysis of 300,000 patients

**DOI:** 10.1038/s41598-023-48235-w

**Published:** 2023-11-27

**Authors:** Jan Oliver Voss, Max Heiland, Robert Preissner, Saskia Preissner

**Affiliations:** 1 Department of Oral and Maxillofacial Surgery, Charité – Universitätsmedizin Berlin, Corporate Member of Freie Universität Berlin, Humboldt-Universität Zu Berlin and Berlin Institute of Health, Augustenburger Platz 1, 13353 Berlin, Germany; 2 Berlin Institute of Health (BIH), Anna-Louisa-Karsch-Straße 2, 10178 Berlin, Germany; 3 Institute of Physiology and Science-IT, Charité – Universitätsmedizin Berlin, Corporate Member of Freie Universität Berlin, Humboldt-Universität Zu Berlin and Berlin Institute of Health, Philippstr. 12, 10115 Berlin, Germany

**Keywords:** Diseases, Risk factors

## Abstract

Postoperative complications following mandibular fracture treatment vary from local wound infections to severe conditions including osteomyelitis and impaired fracture healing. Several risk factors have been associated with the development healing disorders, including fracture localisation, treatment modality and substance abuse. However, limited research on the sex-specific influence of these complications exists. A total of about 300,000 female and male patients with mandibular fractures were examined in two cohorts. After matching for confounders (age, nicotine and alcohol dependence, malnutrition, overweight, anaemia, diabetes, osteoporosis and vitamin D deficiency), two cohorts were compared with propensity-score-matched patients according to outcomes (osteomyelitis, pseudoarthrosis and disruption of the wound) within 1 year after fracture. There were significant differences between female and male patients regarding the occurrence of osteomyelitis (odds ratio [OR] [95% confidence interval]: 0.621 [0.563; 0.686]) and disruption of the wound (OR [95% confidence interval]: 0.703 [0.632; 0.782]). Surprisingly, matching for the expected confounders did not change the results substantially. Sex plays a dominant role in determining the risk stratification for postoperative osteomyelitis and disruption of the wound, after accounting for other potential confounding factors. Additional research is needed to understand the underlying mechanisms and to develop sex-specific strategies to prevent these complications.

## Introduction

Mandibular fractures are common fractures of the facial skeleton. Their occurrence varies not only between different age groups but also across countries and between different time periods^1–4^.The majority (up to 80%) of patients affected by mandibular fractures are men; however, this predominance is equalised in older age groups^5^. Wasicek et al. analyzed the fracture patterns of facial fractures in over 600,000 patients using the National Trauma Data Bank, reporting an overall occurrence of 19% for mandibular fractures^4^. Allareddy et al. reported similar results, providing an epidemiological description of facial fractures in the United States based on a nationally representative, hospital-based emergency department database encompassing over 400,000 patients^6^. The cause, frequency and anatomical distribution of mandibular fractures exhibit significant regional variations, with fractures of the condyle, body and ramus being the most commonly affected areas^7–9^. The treatment options for mandibular fractures include both conservative and various surgical procedures including open reduction and internal fixation and must be adapted to the specific factors of each patient as well as the fracture characteristics itself^10,11^. Dislocated fractures in the tooth-bearing portion are commonly treated according to the basic principles of the Arbeitsgemeinschaft für Osteosynthesefragen (AO) with fracture reduction and internal fixation using osteosynthesis materials^12^. While the majority of fractures show normal/uneventful fracture healing, there is a risk of postoperative wound healing disorder, fixation plate exposure, osteomyelitis and delayed/impaired or even absent fracture healing with pseudarthrosis formation^13,14^. The risk varies depending on the patient population and the associated inclusion criteria^7,13,15,16^. Researchers have associated various factors with severe complications after surgical fracture treatment including the treatment modality, the fracture pattern itself as well as increased time from injury to treatment and patient-specific characteristics, including non-compliance, depression, underlying metabolic diseases (especially diabetes mellitus), removal of a tooth in the fracture gap and substance abuse, contribute to long-term complications^13–15,17–19^. Depending on the severity of impaired bone healing, revision surgery with renewed osteosynthetic treatment, high-dose antibacterial therapy and even bone grafting may be required^20^. Revision surgeries are associated with up to a 32.6% increase in hospital costs, posing an additional burden on both the healthcare system and individual patients^21^. In a large retrospective analysis covering cases of osteomyelitis of various anatomical sites in the United States over 40 years, the annual incidence was significantly higher in male compared with female patients and increase with age (*P* < 0.001)^22^. However, most of the published data regarding a sex-dependent influence on osteomyelitis are for foot and long bones, and little is known about this effect on the mandible^23–27^. Given this lack of knowledge, the aim of the study was to analyse the influence of sex on the development of osteomyelitis, pseudoarthrosis and disruption of the wound after mandibular fractures.

## Results

### Assessment, allocation and matching

We considered a total of 302,575 patients who were diagnosed with mandibular fractures (International Classification of Diseases 10th revision [ICD-10] code S02.6). We grouped the patients according to sex (female vs male). The female cohort included 115,051 patients with a mean ± standard deviation (SD) age of 43.9 ± 24.4 years. The male cohort included 187,524 patients with a mean ± SD age of 34.4 ± 19.7 years. There was a significant difference in age between the sexes (*P* < 0.001). The analyses of the risk factors between the female and male cohort revealed significant differences in nicotine and alcohol dependence, diabetes, overweight, malnutrition, anaemia, vitamin D deficiency and osteoporosis between female and male patients (*P* < 0.001). Table 1 shows the patient characteristics before and after propensity-score matching.

**Table 1:** Patient characteristics before and after propensity-score matching.

	Before matching				After matching			
	Female	Male	*P*	Std. mean difference	Female	Male	*P*	Std. mean difference
Total number of patients	115,051	187,524			96,245	96,245		
Age, mean (years)	43.9	34.4	< 0.001	0.430	39.5	39.3	0.021	0.011
SD	24.4	19.7			23.1	22.7		
Nicotine dependence, n (%)	8211 (7.3%)	8585 (4.8%)	< 0.001	0.088	5491 (5.7%)	6189 (6.4%)	< 0.001	0.030
Alcohol dependence, n (%)	2057 (1.8%)	5135 (2.8%)	< 0.001	0.068	1806 (1.9%)	1880 (2.0%)	0.218	0.006
Diabetes, n (%)	12,714 (11.2%)	11,449 (6.3%)	< 0.001	0.173	8159 (8.5%)	9041 (9.4%)	< 0.001	0.032
Overweight, obesity, + hyperalimentation, n (%)	13,062 (11.5%)	8931 (5.0%)	< 0.001	0.241	7644 (7.9%)	8163 (8.5%)	< 0.001	0.020
Malnutrition, n (%)	2165 (1.9%)	2741 (1.5%)	< 0.001	0.030	1501 (1.6%)	1593 (1.7%)	0.095	0.008
Anaemia, n (%)	14,859 (13.1%)	12,241 (6.8%)	< 0.001	0.213	8914 (9.3%)	10,056 (10.4%)	< 0.001	0.040
Vit. D deficiency, n (%)	9483 (8.4%)	3673 (2.0%)	< 0.001	0.288	3721 (3.9%)	3585 3.7%	0.105	0.007
Osteoporosis, n (%)	10,045 (8.9%)	1546 (0.9%)	< 0.001	0.379	1488 (1.5%)	1546 (1.6%)	0.289	0.005

*Std.* standardised; *Vit.* Vitamin.

Percentage refers to the respective cohorts.

*P*-value refers to the comparison between both cohorts (log-rank test).

### Risk analysis

We performed statistical analysis to compare three outcomes—osteomyelitis, pseudoarthrosis and disruption of the wound—between female and male patients (Tables 2 and 3). Osteomyelitis within 1 year after mandibular fracture occurred in 651 female patients and 1038 male patients. The risk difference was significant (*P* < 0.001, log-rank test). The risk ratio (RR) was 0.624 (95% confidence interval [CI] [0.566; 0.688]) and the odds ratio (OR) was 0.621 (95% CI [0.563; 0.686]).

**Table 2:** Risk difference, risk ratios and odds ratios for osteomyelitis of the female and male cohort after propensity-score matching.

Cohort statistics				
	Number of patients	Number of patients with osteomyelitis	Risk	
Female	95,321	651	0.007	
Male	94,819	1038	0.011	

Risk analysis				
		95% CI	*Z*	*P*
Risk difference	− 0.004	− 0.005, − 0.003	− 9.568	0.0001
Risk ratio	0.624	0.566, 0.688		
Odds ratio	0.621	0.563, 0.686		

The outcome was defined as the occurrence of osteomyelitis within 1 year after mandibular fracture.

*CI* confidence interval.

Note that 924 female patients and 1426 male patients were excluded from the results because they had the outcome prior to the time window.

**Table 3:** Risk difference, risk ratios and odds ratios for disruption of wound of the female and male cohort after propensity-score matching.

Cohort statistics				
	Number of patients	Number of patients with disruption of wound	Risk	
Female	95,587	584	0.006	
Male	95,388	827	0.009	

Risk analysis				
		95% CI	*Z*	*P*
Risk difference	− 0.003	− 0.003, − 0.002	− 6.532	0.0001
Risk ratio	0.705	0.634, 0.783		
Odds ratio	0.703	0.632, 0.782		

The outcome was defined as the occurrence of disruption of the wound within 1 year after mandibular fracture.

*CI* confidence interval.

Note that 658 female patients and 857 male patients were excluded from results because they had the outcome prior to the time window.

For pseudoarthrosis, there was no significant difference between female and male patients (*P* = 0.387). We found pseudoarthrosis in 662 female patients and 694 male patients within 1 year of mandibular fracture. The RR was 0.954 (95% CI [0.858; 1.061]) and the OR was 0.954 (95% CI [0.857; 1.062]).

Disruption of the wound occurred in 584 female patients and 827 male patients within 1 year of mandibular fracture (*P* < 0.0001; Table 3). The RR was 0.705 (95% CI [0.634; 0.783]) and the OR was 0.703 (95% CI [0.632; 0.782]).

A more in-depth analysis was conducted based on ICD subcodes (Supplementary Table 1). In this context, the examination of individual subcodes (S02.6–S02.69) showed no significant differences in odds ratios compared to the overarching code S02.6.

## Discussion

Complications following treatment of mandibular fractures range from mild wound infections and wound healing disorders, wound dehiscence with exposure of fixation material, severe bone infections/osteomyelitis and disturbed/delayed bone healing to failure of fracture healing and the development of pseudarthrosis^28–30^. While minor complications can be treated conservatively, revision surgery with refixation might be indicated in cases of severe impaired fracture healing^31^. In a retrospective study by Steffen et al. the main indications for revision surgery with refixation were osteomyelitis (52.9%) and non-union (41.2%) in patients with mandibular fractures^32^. Different factors have been associated with complications after fracture treatment, including smoking and alcohol abuse, increased time from injury to treatment, mandibular fracture severity, treatment modality and tooth extraction^13,17,26,27^. Currently, there is limited data on sex-specific considerations for complications like osteomyelitis following mandibular fracture treatment. Gordon et al. conducted a case–control study on patients who underwent mandibular fracture repair, discovering a higher rate of postoperative inflammatory complications (POIC), including osteomyelitis, among male patients. However, in their bivariate analysis, gender did not exert a significant influence^26^. Lukošiūnas and colleagues analyzed data from patients who developed osteomyelitis after mandibular fracture treatment and compared background factors of complications with a control group. They did not observe a gender-specific effect on the development of osteomyelitis. However, in their logistic regression analysis, the authors identified several significant factors in the development of osteomyelitis in fractured mandibles. These included factors such as immunity dysfunction, oral microflora, presence of caries-affected or intact teeth at the fracture line, mobility of bone fragments, inadequate repositioning, and delayed fixation of bone fragments after trauma^33^.

In a comprehensive retrospective study analysing 760 cases of osteomyelitis across various anatomical sides, the incidence was higher for men than for women and increased with age (*P* < 0.001). In this retrospective study encompassing Olmsted County, Minnesota residents, only 19% of osteomyelitis cases were linked to traumatic origins. Additionally, craniofacial sites accounted for merely about 5% of all anatomical locations in the study, rendering the interpretation of data on mandibular osteomyelitis less reliable^22^.

To evaluate the sex-specific effect on the development of postoperative wound dehiscence, osteomyelitis and pseudarthrosis of the mandible following fracture treatment, we performed one-to-one matching of male and female patients based on similar covariate distributions, including alcohol and nicotine dependence; diabetes mellitus; malnutrition, overweight, obesity and hyperalimentation; osteoporosis; anaemia; and vitamin D deficiency. Interestingly, there were significant differences in all of these parameters before matching between men and women. Moreover, after matching for confounders, a significant difference between male and female patients regarding the development of postoperative disruption of the wound and the development of osteomyelitis was found. There are several potential reasons for the higher incidence of osteomyelitis following mandibular fracture treatment in men. Males generally exhibit a greater quantity of cortical bone in the mandible, highlighting sex-related differences^34^. Studies on chronic osteomyelitis reported the invasion and persistence of Staphylococcus aureus in the canaliculi of live cortical bone^35,36^, which may serve as a mechanism for promoting persistent and chronic infection, potentially restricting immune cell access^37^. One factor might be the higher rate of nicotine abuse in men, which has generally been attributed to a higher complication rate^30^. In their review of the potentially modifiable patient factors that could affect mandibular fracture complications, Ahmed et al. identified smoking as the most common potentially modifiable factor (OR 4.04–8.09)^38^. To exclude differences in the prevalence of nicotine abuses between both sexes, we conducted confounder matching including nicotine abuse. However, differences in smoking habits have been reported with higher pack-years of smoking and number of cigarettes per day in men^39^. Interestingly, Radabaugh et al.^40^ analysed patient compliance following mandibular fracture repair and concluded that current tobacco use is negatively associated with patient compliance. Another aspect is the dominance of interpersonal violence in mandibular fractures in men, leading to a different fracture pattern with a higher susceptibility for osteomyelitis compared with women with mandibular fractures^15,19,41^. In general, sex-related differences in lifestyle may also affect health status and therefore the prevalence of pre-existing conditions^42^. In this regard, social and behavioural characteristics are key factors related to the sex gap in mortality^43^.

Studies in the field of osteoimmunology provide information regarding the modulating effect of the innate and adaptive immune system on bone resorption during inflammations^44^. In men and women, the development and functioning of the immune system are affected in distinct ways by various environmental factors, such as the nutrition status and the composition of the microbiome. These sex-based immunological differences contribute to variations in the incidence and susceptibility to infectious diseases^45^. Interestingly, different human and animal studies focused on fracture healing in long bones have reported impaired bone healing more often in women^46,47^, suggesting possible sex-based differences in bone healing in general^48^. In a large patient database analysis of more than 300.000 fractures in 18 bone, ORs for non-union fractures were significantly increased for different risk factors including male gender (OR 1.21; 95%, CI 1.16–1.25)^49^. However, there are several differences between mandibular and long bone fractures. First, mandibular fracture wounds could be contaminated by bacteria of the oral cavity. Second, there is different underlying embryonic bone development/formation: endochondral bone formation in long bones and intramembranous bone formation of the mandible.

Limitations of this study are that the data analysis is conducted on a large patient database, which might lead to unexpected associations as well as to a selection bias^50^. It is important to note that the associations identified in this study do not imply causation. Furthermore, the TriNetX database did not include information on the time elapsed between injury and treatment, potentially introducing an unobserved confounding variable. Several studies have analysed the effects of a treatment delay, reporting conflicting results, which might be attributed to a lack of consensus on the definitions of "early" versus "delayed" intervention^18,27,28,33^.

## Conclusion and future perspectives

Sex plays a dominant role in determining the risk stratification for postoperative osteomyelitis and disruption of the wound, after accounting for other potential confounding factors.Sex-specific treatment recommendations should be considered to account for the sex-specific risk for the development of osteomyelitis. A possible recommendation is prolonged peri- and postoperative antibacterial therapy in men with the corresponding risk profile and risk factors as well as an extended follow-up observation.

## Patients and methods

### Data acquisition and inclusion and exclusion criteria

We used TriNetX, a global federated health research network providing access to statistics on electronic medical records (diagnoses, procedures, medications, laboratory values, genomic information) from patients in large Healthcare Organizations predominately. As a federated network, TriNetX received a waiver from Western IRB since only aggregated counts, statistical summaries of de-identified information, but no protected health information is received, and no study-specific activities are performed in retrospective analyse.

This retrospective study is exempt from informed consent. The data reviewed is a secondary analysis of existing data, does not involve intervention or interaction with human subjects, and is de-identified per the de-identification standard defined in Section §164.514(a) of the HIPAA Privacy Rule. The process by which the data is de-identified is attested to through a formal determination by a qualified expert as defined in Section §164.514(b)(1) of the HIPAA Privacy Rule. This formal determination by a qualified expert refreshed on December 2020. The TriNetX network was accessed on June 23rd, 2023. The query was run on the platform with a group of 81 health care organisations (HCOs). The database was searched for electronic medical records up to 20 years before the access date for patients with mandibular fractures according to the ICD-10 (International Statistical Classification of Diseases and Related Health Problems) code S02.6. Based on the subdivision of the S02.6 code (S02.60–S02.69), a subgroup analysis of the individual codes is displayed in Supplements (Supplementary Table 1).

**Figure 1:**
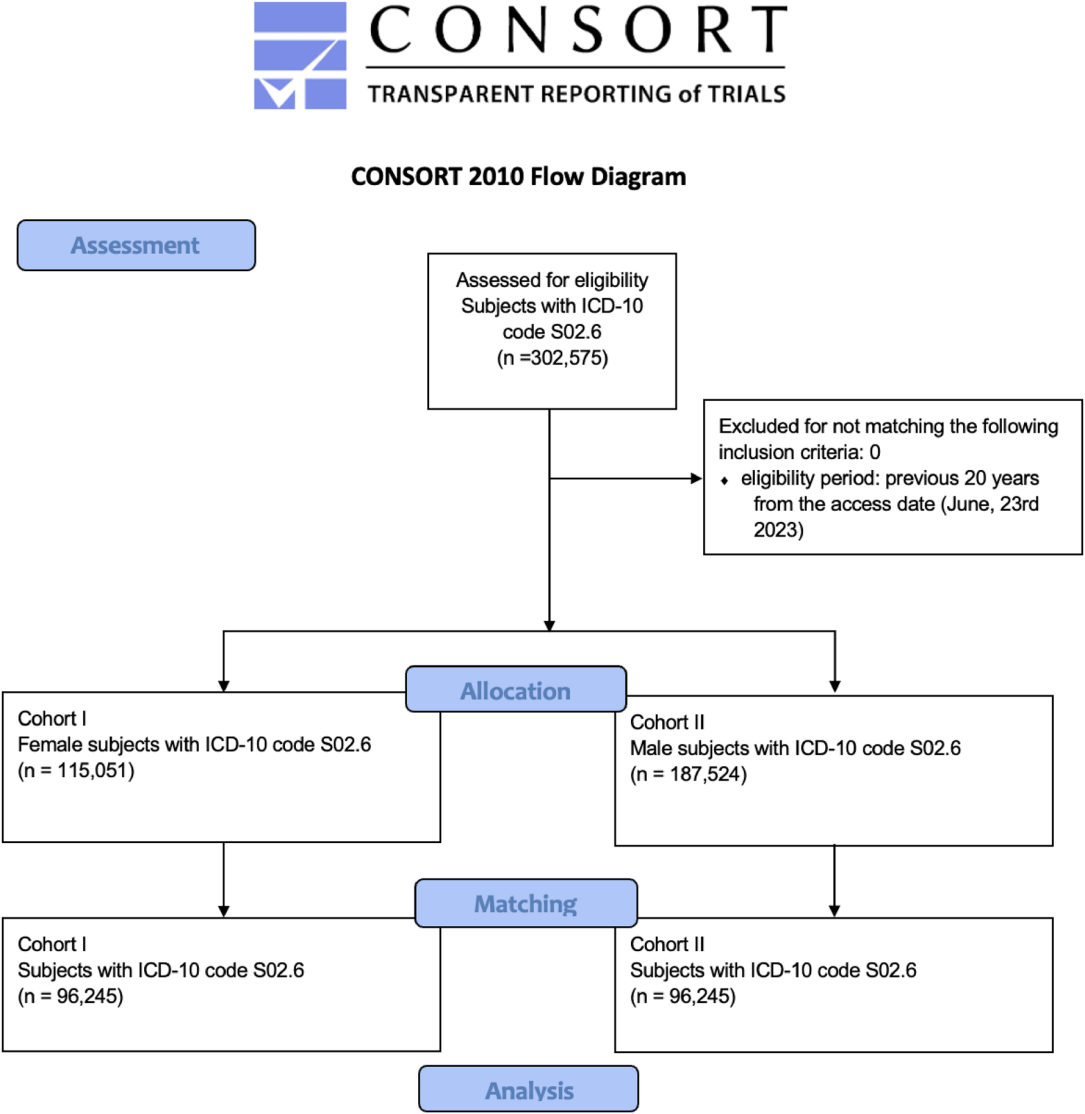
Modified Consolidated Standards of Reporting Trials (CONSORT) flow chart.

Figure 1 displays a modified Consolidated Standards of Reporting Trials (CONSORT) flow chart. We grouped patients according to sex (female vs male). Before matching, there were 115,051 patients from 71 HCOs in the female cohort (cohort I) and 187,524 patients from 73 HCOs in the male cohort (cohort II). We applied propensity-score matching to reduce confounding variables and to ensure the groups were based on similar covariate distributions. We performed one-to-one matching for alcohol and nicotine dependence; diabetes mellitus; malnutrition; overweight, obesity and hyperalimentation; osteoporosis, anaemia; and vitamin D deficiency. After matching, each cohort had 96,245 patients.

### Data analysis

We defined the index event as the day of the mandibular fracture; the observation period was 1 year after the mandibular fracture. We defined the outcomes as osteomyelitis (ICD-10 code M86), pseudoarthrosis after fusion (ICD-10 code M96.0) and disruption of the wound (ICD-10 code T81.3). We excluded patients with the above-mentioned outcomes prior to the index event from the analyses. We conducted propensity-score matching using a nearest neighbor greedy matching algorithm with a caliper of 0.25 times the standard deviation. Statistical analysis included a risk analysis. We calculated the risk difference, RR, OR—each with a 95% CI—and performed the log-rank test to compare treatment outcomes between the two groups. We considered *P* < 0.05 to be statistically significant.

### Ethics approval and consent to participate

Administrative access to the database was granted by TriNetX. All methods were carried out in accordance with relevant guidelines and regulations. All Healthcare Organizations (HCOs) from which data were transmitted to TriNetX obtained written informed consent from all patients and/or their legal guardians. Experimental protocols and ethical approval were approved from the appropriate authorities. TriNetX is compliant with the Health Insurance Portability and Accountability Act (HIPAA), the US federal law which protects the privacy and security of healthcare data. TriNetX is certified to the ISO 27001:2013 standard and maintains an Information Security Management System (ISMS) to ensure the protection of the healthcare data it has access to and to meet the requirements of the HIPAA Security Rule. Any data displayed on the TriNetX Platform in aggregate form, or any patient-level data provided in a data set generated by the TriNetX Platform, only contains de-identified data as per the de-identification standard defined in Section §164.514(a) of the HIPAA Privacy Rule. The process by which the data are de-identified is attested to through a formal determination by a qualified expert as defined in Section §164.514(b) [1] of the HIPAA Privacy Rule. This formal determination by a qualified expert, refreshed in December 2020, supersedes the need for TriNetX’s previous waiver from the Western Institutional Review Board (IRB). The TriNetX network contains data provided by participating HCOs, each of which represents and warrants that it has all necessary rights, consents, approvals, and authority to provide the data to TriNetX under a Business Associate Agreement (BAA), so long as their name remains anonymous as a data source and their data are utilized for research purposes. The data shared through the TriNetX Platform are attenuated to ensure that they do not include sufficient information to facilitate the determination of which HCO contributed which specific information about a patient (https://trinetx.com/trinetx-publication-guidelines/). Access to the database is closed.

### Supplementary Information

Supplementary Table 1.

## Data Availability

To gain access to the data in the TriNetX research network, a request can be made to TriNetX (https://live.trine tx.com), but costs may be incurred, a data sharing agreement would be necessary, and no patient identifiable information can be obtained. Data is available on reasonable request from the corresponding author.

## References

[1] Gassner R, Tuli T, Hachl O, Rudisch A, Ulmer H (2003). Cranio-maxillofacial trauma: A 10 year review of 9,543 cases with 21,067 injuries. J. Craniomaxillofac. Surg..

[2] Lee K (2012). Global trends in maxillofacial fractures. Craniomaxillofac. Trauma Reconstr..

[3] Roden KS (2012). Changing characteristics of facial fractures treated at a regional, level 1 trauma center, from 2005 to 2010: An assessment of patient demographics, referral patterns, etiology of injury, anatomic location, and clinical outcomes. Ann. Plast. Surg..

[4] Wasicek PJ (2019). Contemporary characterization of injury patterns, initial management, and disparities in treatment of facial fractures using the national trauma data bank. J. Craniofac. Surg..

[5] Adik K (2023). Trends in mandibular fractures in the USA: A 20-year retrospective analysis. Dent. Traumatol..

[6] Allareddy V, Allareddy V, Nalliah RP (2011). Epidemiology of facial fracture injuries. J. Oral Maxillofac. Surg..

[7] Bormann KH (2009). Five-year retrospective study of mandibular fractures in Freiburg, Germany: Incidence, etiology, treatment, And Complications. J. Oral Maxillofac. Surg..

[8] Ellis E, Moos KF, El-Attar AT (1985). Years of mandibular fractures: An analysis of 2,137 cases. Oral Surg. Oral Med. Oral Pathol..

[9] Eskitascioglu T (2013). Fractures of the mandible: A 20-year retrospective analysis of 753 patients. Ulus Travma Acil Cerrahi Derg.

[10] Nasser M (2013). Interventions for the management of mandibular fractures. Cochrane Database Syst. Rev..

[11] Panesar K, Susarla SM (2021). Mandibular fractures: Diagnosis and management. Semin. Plast. Surg..

[12] Ehrenfeld M, Manson PN, Prein J (2012). Principles of Internal Fixation of the Craniomaxillofacial Skeleton.

[13] Hsieh TY (2019). Risk factors associated with complications after treatment of mandible fractures. Jama Facial Plast. Surg..

[14] Serena-Gomez E, Passeri LA (2008). Complications of mandible fractures related to substance abuse. J. Oral Maxillofac. Surg..

[15] Mathog RH, Toma V, Clayman L, Wolf S (2000). Nonunion of the mandible: An analysis of contributing factors. J. Oral Maxillofac. Surg..

[16] Giordano AM, Foster CA, Boies LR, Maisel RH (1982). Chronic osteomyelitis following mandibular fractures and its treatment. Arch. Otolaryngol..

[17] Furr AM, Schweinfurth JM, May WL (2006). Factors associated with long-term complications after repair of mandibular fractures. Laryngoscope.

[18] Lander DP (2021). The impact of treatment delay on malunion and nonunion after open reduction of mandible fractures. Facial Plast. Surg. Aesthet. Med..

[19] Christensen BJ, Mercante DE, Neary JP, King BJ (2017). Risk factors for severe complications of operative mandibular fractures. J. Oral Maxillofac. Surg..

[20] Ostrander BT (2018). Contemporary management of mandibular fracture nonunion-a retrospective review and treatment algorithm. J. Oral Maxillofac. Surg..

[21] Lee KC, Chuang SK, Koch A (2019). The healthcare cost of mandibular nonunions. J. Craniofac. Surg..

[22] Kremers HM (2015). Trends in the epidemiology of osteomyelitis: A population-based study, 1969 to 2009. J. Bone Jt. Surg. Am..

[23] Garcia Del Pozo E, Collazos J, Carton JA, Camporro D, Asensi V (2018). Factors predictive of relapse in adult bacterial osteomyelitis of long bones. BMC Infect. Dis..

[24] Tice AD, Hoaglund PA, Shoultz DA (2003). Risk factors and treatment outcomes in osteomyelitis. J. Antimicrob. Chemother..

[25] Slyamova G, Gusmanov A, Batpenov A, Kaliev N, Viderman D (2022). Risk factors for postoperative osteomyelitis among patients after bone fracture: A matched case-control study. J. Clin. Med..

[26] Gordon PE, Lawler ME, Kaban LB, Dodson TB (2011). Mandibular fracture severity and patient health status are associated with postoperative inflammatory complications. J. Oral Maxillofac. Surg..

[27] Stone IE, Dodson TB, Bays RA (1993). Risk factors for infection following operative treatment of mandibular fractures: A multivariate analysis. Plast. Reconstr. Surg..

[28] Lee UK, Rojhani A, Herford AS, Thakker JS (2016). Immediate versus delayed treatment of mandibular fractures: A stratified analysis of complications. J. Oral Maxillofac. Surg..

[29] Malanchuk VO, Kopchak AV (2007). Risk factors for development of infection in patients with mandibular fractures located in the tooth-bearing area. J. Craniomaxillofac. Surg..

[30] Gutta R (2014). Outcomes of mandible fracture treatment at an academic tertiary hospital: A 5-year analysis. J. Oral Maxillofac. Surg..

[31] Chen CL, Zenga J, Patel R, Branham G (2018). Complications and reoperations in mandibular angle fractures. Jama Facial Plast. Surg..

[32] Steffen, C. *et al.* Revision surgery with refixation after mandibular fractures. *Craniomaxillofac Trauma Reconstr* **0**, 19433875231179318 10.1177/19433875231179318 10.1177/19433875231179318 PMC11425749 39345950

[33] Lukosiunas A, Kubilius R, Sabalys G, Keizeris T, Sakavicius D (2011). An analysis of etiological factors for traumatic mandibular osteomyelitis. Medicina (Kaunas).

[34] Matsuura T (2014). Sex-related differences in cortical and trabecular bone quantities at the mandibular molar. J. Hard Tissue Biol..

[35] De Mesy Bentley KL, Macdonald A, Schwarz EM, Oh I (2018). Chronic osteomyelitis with staphylococcus aureus deformation in submicron canaliculi of osteocytes: A case report. Jbjs Case Connect.

[36] De Mesy Bentley KL (2017). Evidence of staphylococcus aureus deformation, proliferation, and migration in canaliculi of live cortical bone in murine models of osteomyelitis. J. Bone Miner. Res..

[37] Hofstee MI (2020). Current concepts of osteomyelitis: From pathologic mechanisms to advanced research methods. Am. J. Pathol..

[38] Ahmed A, Wu E, Sarai R, Williams R, Breeze J (2022). Potentially modifiable patient factors in mandible fracture complications: A systematic review and meta-analysis. Br. J. Oral Maxillofac. Surg..

[39] Peters SA, Huxley RR, Woodward M (2014). Do smoking habits differ between women and men in contemporary western populations? Evidence from half a million people in the Uk Biobank Study. BMJ Open.

[40] Radabaugh JP, Horn AV, Chan SA, Shelton JM, Gal TJ (2017). Patient compliance following isolated mandibular fracture repair. Laryngoscope.

[41] Rashid A, Eyeson J, Haider D, Van Gijn D, Fan K (2013). Incidence and patterns of mandibular fractures during a 5-year period in a London teaching hospital. Br. J. Oral Maxillofac. Surg..

[42] Vari R (2016). Gender-related differences in lifestyle may affect health status. Ann Ist Super Sanita.

[43] Rogers RG, Everett BG, Onge JM, Krueger PM (2010). Social, behavioral, and biological factors, and sex differences in mortality. Demography.

[44] Kumar G, Roger PM (2019). From crosstalk between immune and bone cells to bone erosion in infection. Int. J. Mol. Sci..

[45] Klein SL, Flanagan KL (2016). Sex differences in immune responses. Nat. Rev. Immunol..

[46] Mehta M, Duda GN, Perka C, Strube P (2011). Influence of gender and fixation stability on bone defect healing in middle-aged rats: A pilot study. Clin. Orthop. Relat. Res..

[47] Parker MJ, Raghavan R, Gurusamy K (2007). Incidence of fracture-healing complications after femoral neck fractures. Clin. Orthop. Relat. Res..

[48] Kurapaty SS, Hsu WK (2022). Sex-based difference in bone healing: A review of recent pre-clinical literature. Curr. Rev. Musculoskelet. Med..

[49] Zura R (2016). Epidemiology of fracture nonunion in 18 human bones. Jama Surg..

[50] Topaloglu U, Palchuk MB (2018). Using a federated network of real-world data to optimize clinical trials operations. Jco Clin. Cancer Inform..

