# Supplementary material for: The risk of osteomyelitis after mandibular fracture is doubled in men versus women: analysis of 300,000 patients
Source: Sci Rep. 2023 Nov 27;13:20871. doi: 10.1038/s41598-023-48235-w (PMC10682452; doi:10.1038/s41598-023-48235-w)
Supplement: Supplementary file 1 — Supplementary Table 1. [file 41598_2023_48235_MOESM1_ESM.docx]

**Supplementary Table 1 Detailed overview of the odds ratios of the subcodes S02.60-S02.69 in relation to postoperative complications.**

|  |  | **ICD-Codes** | | | | | | | | |
| --- | --- | --- | --- | --- | --- | --- | --- | --- | --- | --- |
|  |  | **S02.6** | **S02.61** | **S02.62** | **S02.63** | **S02.64** | **S02.65** | **S02.66** | **S02.67** | **S02.69** |
| **Postoperative complication** |  | **Fracture of mandible** | **Fracture of condylar process of mandible** | **Fracture of subcondylar process of mandible** | **Fracture of coronoid process of mandible** | **Fracture of ramus of mandible** | **Fracture of angle of mandible** | **Fracture of symphysis of mandible** | **Fracture of alveolus of mandible** | **Fracture of multiple sites** |
| Osteomyelitis | OR 95% CI | 0.62 (0.56,0.69) | 0.6 (0.53,0.67) | 0.61 (0.55,0.69) | 0.59 (0.52,0.66) | 0.57 (0.51,0.64) | 0.58 (0.52,0.66) | 0.572 (0.51,0.64) | 0.59 (0.52,0.6) | 0.58 (0.52,0.66) |
| Pseudoarthrosis | OR 95% CI | 0.95 (0.86,1.06) | 0.89 (0.8,0.99) | 0.87 (0.78,0.97) | 0.83 (0.75,0.92) | 0.86 (0.78,0.96) | 0.94 (0.84,1.04) | 0.91 (0.82,1.02) | 0.85 (0.76,0.94) | 0.9 (0.8,0.99) |
| Disruption of wound | OR 95% CI | 0.7 (0.63,0.78) | 0.68 (0.6,0.78) | 0.71 (0.62,0.82) | 0.71 (0.62,0.81) | 0.7 (0.61,0.8) | 0.64 (0.56,0.73) | 0.75 (0.66,0.85) | 0.7 (0.61,0.81) | 0.7 (0.62,0.8) |

CI = confidence interval

ICD = International Statistical Classification of Diseases and Related Health Problems
